# Supplementary figures and images for: Exploration adhesion properties of Liquorilactobacillus and Lentilactobacillus isolated from two different sources of tepache kefir grains
Source: PLoS One. 2024 Feb 7;19(2):e0297900. doi: 10.1371/journal.pone.0297900 (PMC10849267; doi:10.1371/journal.pone.0297900)

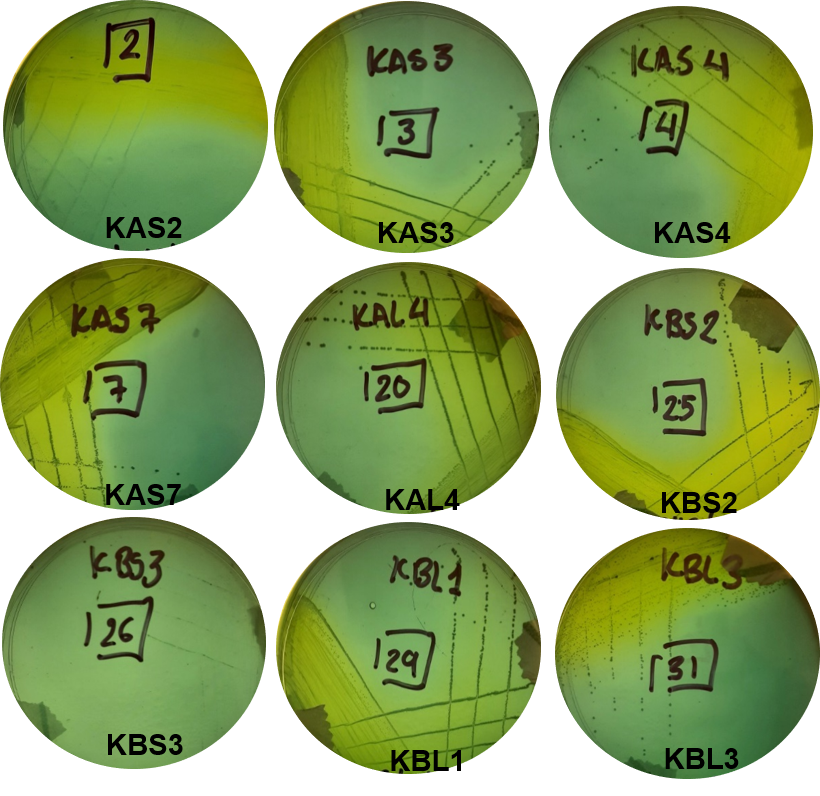

Supplement: S1 Fig — (TIF) [file pone.0297900.s001.tif]

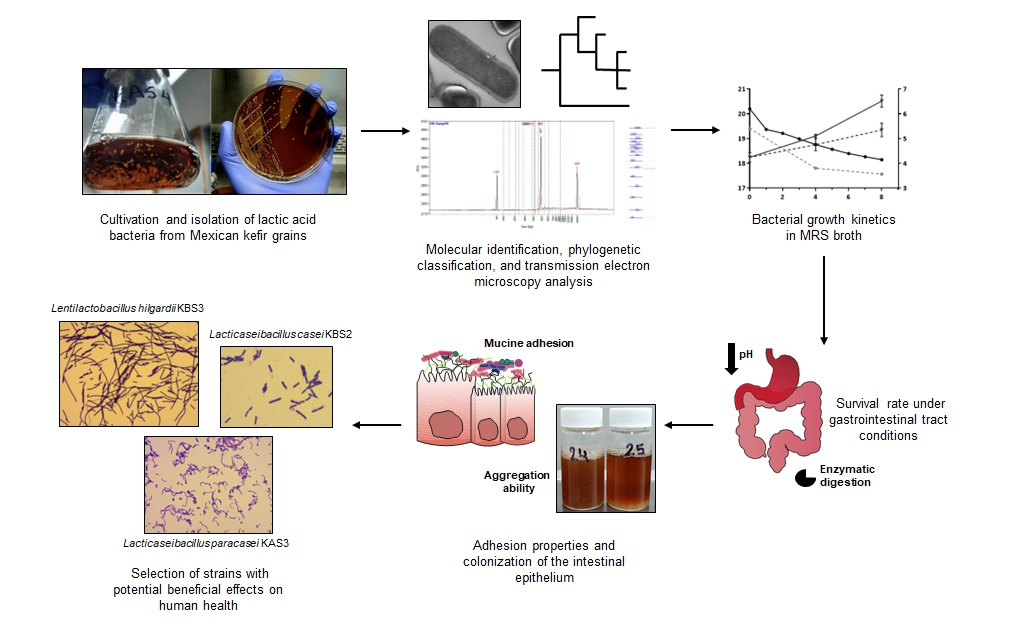

Supplement: S1 Graphical abstract — (TIF) [file pone.0297900.s005.tif]
