# Supplementary material for: Exploration adhesion properties of Liquorilactobacillus and Lentilactobacillus isolated from two different sources of tepache kefir grains
Source: PLoS One. 2024 Feb 7;19(2):e0297900. doi: 10.1371/journal.pone.0297900 (PMC10849267; doi:10.1371/journal.pone.0297900)
Supplement: S1 Table — (PDF) [file pone.0297900.s002.pdf]

S1 Table. Biochemical profile evaluated by API 50 CHL kit assay.

Compound/derivative	Strains								
	KAS2	KAS3	KAS4	KAS7	KAL4	KBS2	KBS3	KBL1	KBL3
Glycerol	-	-	-	-	-	-	-	-	-
Erythritol	-	-	-	-	-	-	-	-	-
D-Arabinose	-	-	-	-	-	-	-	-	-
L-Arabinose	-	-	-	-	-	-	-	-	-
D-Ribose	+	+	+	-	-	+	+	-	+
D-Xylose	-	-	-	-	-	-	+	-	-
L-Xylose	-	-	-	-	-	-	-	-	-
D-Adonitol	-	-	+	-	-	-	-	-	-
Methyl-D-xylopyraniside	-	-	-	-	-	-	-	-	-
D-Galactose	+	+	+	-	-	+	-	-	+
D-Glucose	+	+	+	+	+	+	-	+	+
D-Fructose	+	+	+	+	+	+	+	+	+
D-Mannose	+	-	+	+	+	+	-	+	+
L-Sorbose	-	+	+	+	+	-	-	+	-
L-Rhamnose	-	-	-	-	-	-	-	-	+

Dulcitol	+	-	-	-	-	-	-	-	-
Inositol	-	-	-	-	-	+/-	-	-	-
D-Mannitol	-	+/-	+	+	+	+	-	+	+
D-Sorbitol	+/-	+	+	-	-	-	-	-	-
Methyl-D-mannopyranoside	-	-	-	-	-	-	-	-	-
Methyl-D-glucopyranoside	+/-	-	-	+	+	+	-	+/-	-
N-acetylglucosamine	+	+	+	+	+	+	-	+/-	+
Amygdaline	-	+	+	-	-	-	-	-	+/-
Arbutine	+	+	+	+	+	+	-	+	+
Esculine	+	+	+	+	+	+	-	+	+
Salicine	+	+	+	+	+	+	-	+	+
D-Celiobiose	+	+	+	-	-	+	-	-	+
D-Maltose	+/-	-	+	-	-	+	-	-	+
D-Lactose	-	-	+	-	-	-	-	-	+
D-Melibiose	-	-	-	-	-	-	-	-	-
D-Saccharose	+	+	+	+	+	+	+	+	+
D-Trehalose	+	+	+	+	+	+	-	+	+

Inuline	-	-	-	-	-	+	-	-	+
D-Melezitose	+	+	+	-	-	+	+	-	+
D-Raffnose	-	-	-	-	-	-	-	-	-
Amidon	-	-	-	-	-	-	-	-	-
Glycogen	-	-	-	-	-	-	-	-	-
Xylitol	-	-	-	-	-	-	-	-	-
Gentiobiose	-	-	-	+	+/-	+	-	+/-	+
D-Turanose	+	+	+	-	-	+	-	-	+
D-Lyxose	-	-	+	-	-	-	-	-	-
D-Tagatose	+	+	+	-	-	+	-	-	+
D-Fucose	-	-	-	-	-	-	-	-	-
L-Fucose	-	-	-	-	-	-	-	-	-
D-Arabitol	-	-	-	-	-	-	-	-	-
L-Arabitol	-	-	-	-	-	-	-	-	-
Potassium gluconate	+	+	+	-	-	-	-	-	+
2-Ceto-gluconate	-	-	-	-	-	-	-	-	-
5-Ceto-gluconate	-	-	-	-	-	-	-	-	-

+, positive; -, negative; +/-, inconclusive.
