# Supplementary material for: Exploration adhesion properties of Liquorilactobacillus and Lentilactobacillus isolated from two different sources of tepache kefir grains
Source: PLoS One. 2024 Feb 7;19(2):e0297900. doi: 10.1371/journal.pone.0297900 (PMC10849267; doi:10.1371/journal.pone.0297900)
Supplement: S2 Table — (PDF) [file pone.0297900.s003.pdf]

S2 Table. Bacterial identification by ABIS online software, based on API 50CHL test.

Strain	Identified gender	Similarity (%)
KAS2	*Lacticaseibacillus paracasei subs. paracasei*	92
KAS3	*Lacticaseibacillus paracasei subs. paracasei*	91.9
KAS4	*Lacticaseibacillus paracasei subs. paracasei*	97.4
KAS7	*Liquorilactobacillus oeni*	92.4
KAL4	*Liquorilactobacillus oeni*	92.4
KBS2	*Lacticaseibacillus paracasei subs. paracasei*	91.9
KBS3	*Levilactobacillus paucivorans*	96.1
KBL1	*Liquorilactobacillus oeni*	92.2
KBL3	*Lacticaseibacillus paracasei subs. paracasei*	94.9

ABIS online - Advanced Bacterial Identification Software, ABIS online - Advanced Bacterial Identification Software is a laboratory tool for bacterial identification, based on morpho-biochemical characters, cultural characteristics, growth conditions, ecology and pathogenicity data.
