# Supplementary material for: Exploration adhesion properties of Liquorilactobacillus and Lentilactobacillus isolated from two different sources of tepache kefir grains
Source: PLoS One. 2024 Feb 7;19(2):e0297900. doi: 10.1371/journal.pone.0297900 (PMC10849267; doi:10.1371/journal.pone.0297900)
Supplement: S1 File — (PDF) [file pone.0297900.s004.pdf]

## Sequencing results on Novaseq 250 bp paired-end sequencing equipment

| Sample | RawPE | Combined | Qualified | Nochime | Base(nt) | Avglen(nt) | GC | Q20 | Q30 | Effective% |
|---|---|---|---|---|---|---|---|---|---|---|
| A1 | 161482 | 159344 | 155357 | 152313 | 65247992 | 428.38 | 52.66% | 98.16% | 94.16% | 94.32% |
| A2 | 151300 | 149274 | 145346 | 143278 | 61416917 | 428.66 | 52.53% | 98.15% | 94.18% | 94.70% |
| A3 | 127374 | 125483 | 121920 | 120433 | 51637338 | 428.76 | 52.73% | 98.06% | 94.01% | 94.55% |
| A4 | 161733 | 158673 | 153170 | 151020 | 64762404 | 428.83 | 50.59% | 97.53% | 92.28% | 93.38% |
| A5 | 158490 | 155502 | 150075 | 148486 | 63680358 | 428.86 | 50.46% | 97.45% | 92.07% | 93.69% |
| A6 | 137637 | 135812 | 132298 | 131286 | 56297769 | 428.82 | 52.70% | 98.20% | 94.31% | 95.39% |
| A7 | 90159 | 88814 | 86683 | 85420 | 36609280 | 428.58 | 52.51% | 98.43% | 94.74% | 94.74% |
| A8 | 144980 | 142269 | 137305 | 132595 | 56849855 | 428.75 | 50.68% | 97.54% | 92.34% | 91.46% |
| A9 | 165963 | 163784 | 159598 | 156946 | 67165448 | 427.95 | 52.61% | 98.17% | 94.21% | 94.57% |

## A1 (KAS2)

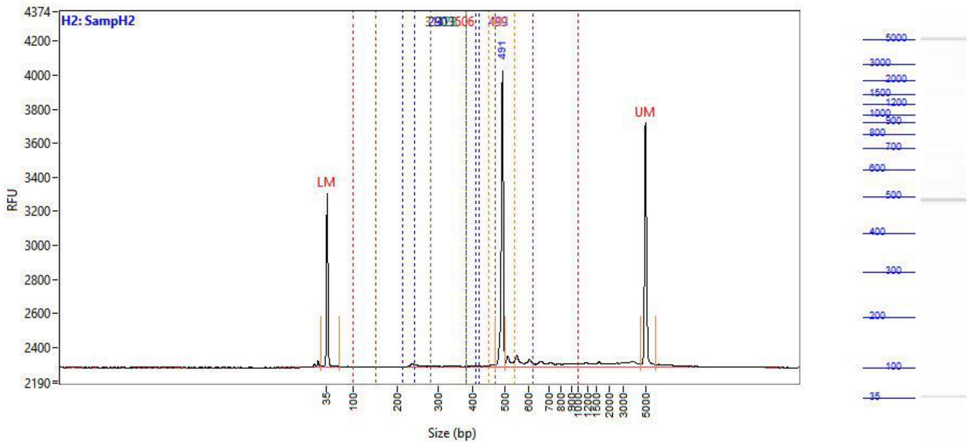


## A2 (KAS3)

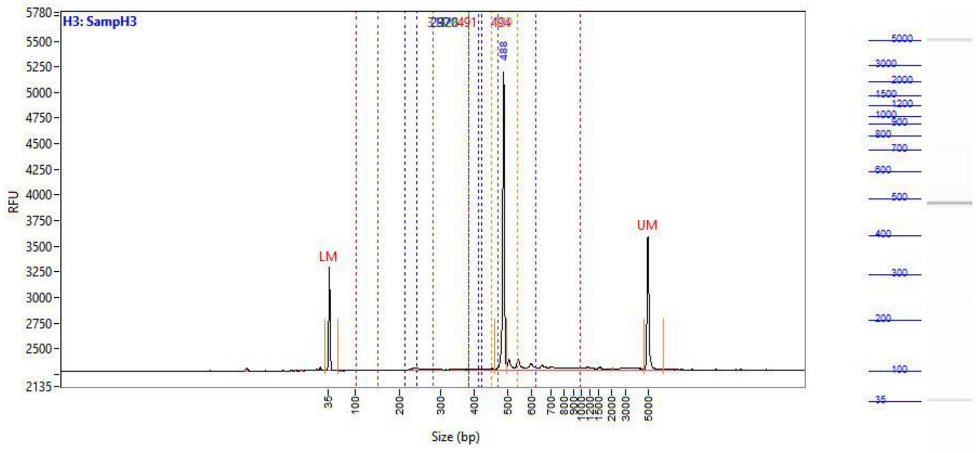


A3 (KAS4)

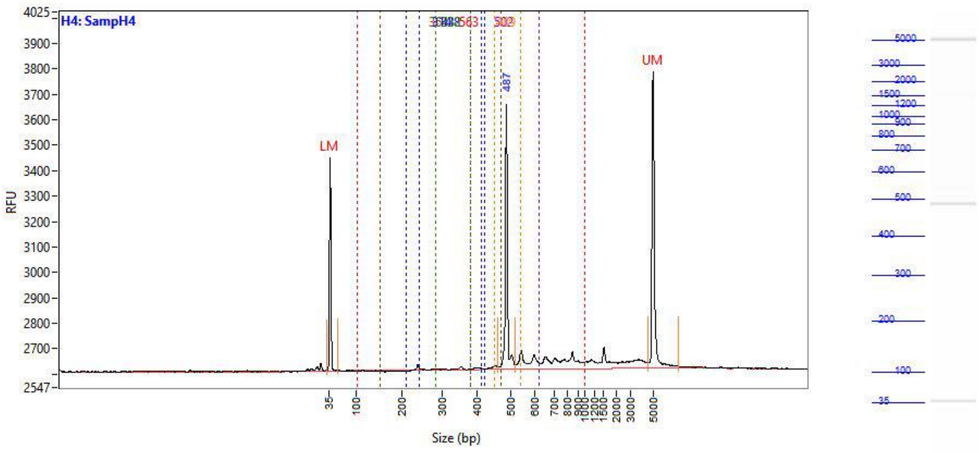


A4 (KAS7)

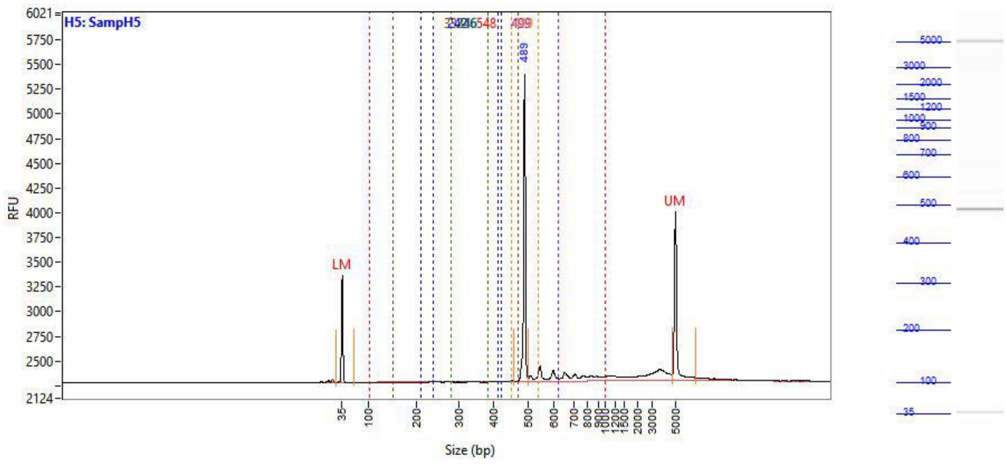


A5 (KAL4)

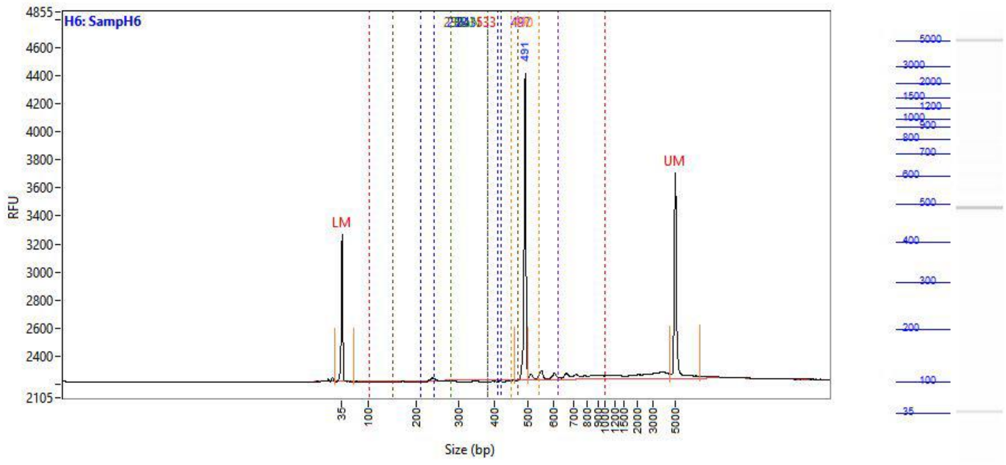

## A6 (KBS2)

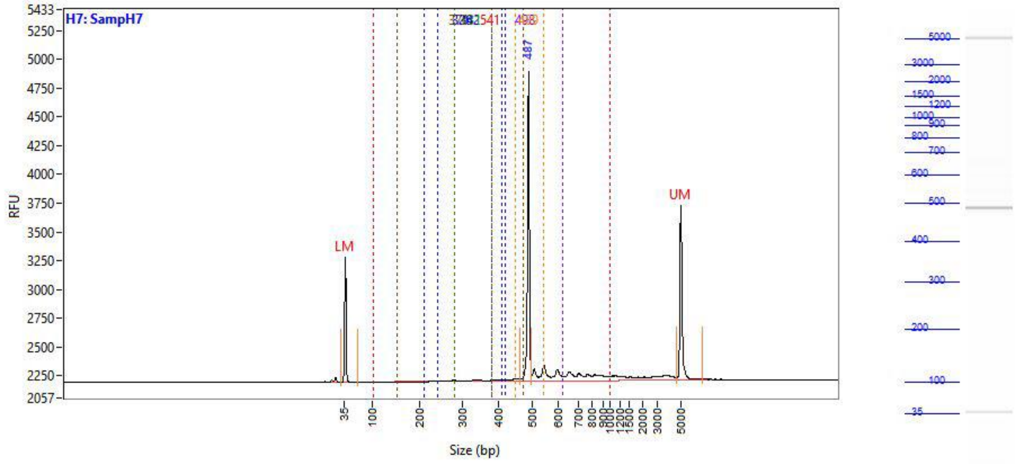


## A7 (KBS3)

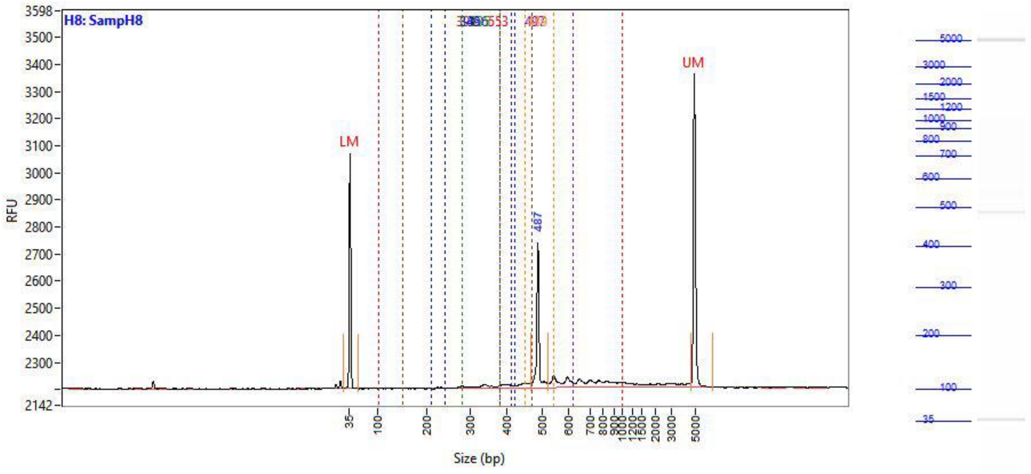


## A8 (KBL1)

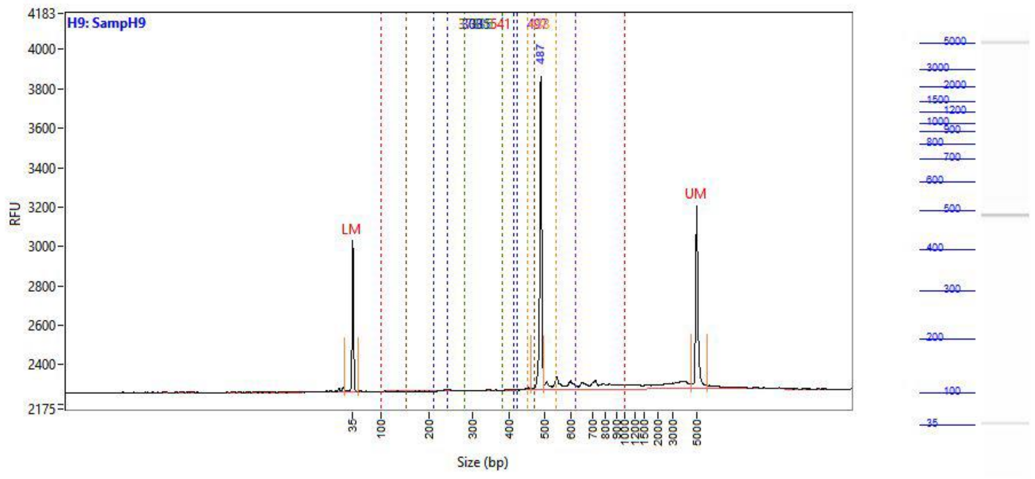
H9: SampH9
RFU
Size (bp)
LM
UM
487

A9 (KBL3)

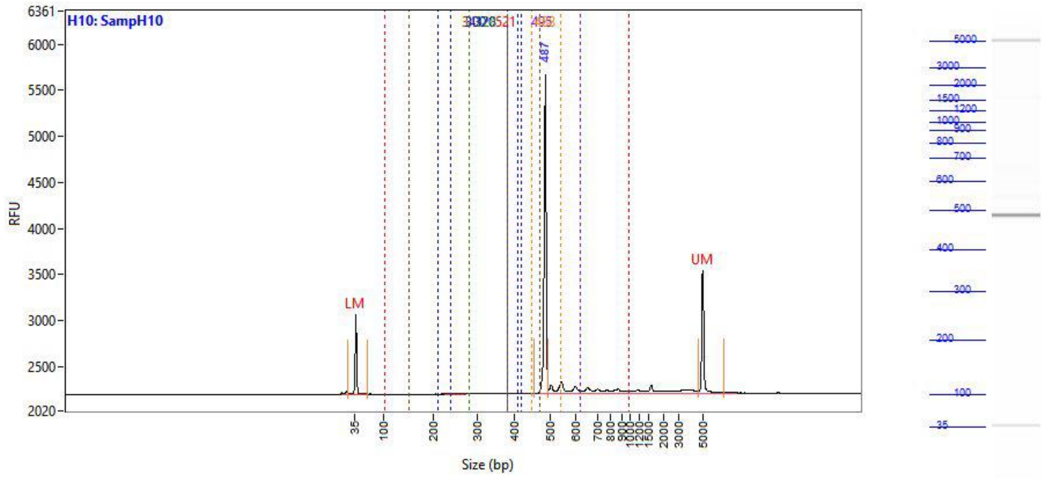
H10: SampH10
RFU
Size (bp)
LM
UM
487
